# Effect of Cr on Microstructure and Properties of WVTaTiCr*_x_* Refractory High-Entropy Alloy Laser Cladding

**DOI:** 10.3390/ma16083060

**Published:** 2023-04-13

**Authors:** Zhaomin Xu, Zhiping Sun, Cheng Li, Zhiming Wang

**Affiliations:** School of Mechanical Engineering, Qilu University of Technology (Shandong Academy of Sciences), Jinan 250353, China

**Keywords:** refractory high-entropy alloys, laser cladding, microstructure, mechanical properties, oxidation resistance

## Abstract

WVTaTiCr*_x_* (*x* = 0, 0.25, 0.5, 0.75, 1) refractory high-entropy alloy coatings were prepared on a 42-CrMo steel plate using laser cladding. The purpose of this work is to investigate the effect of the Cr content on the microstructure and properties of the WVTaTiCr*_x_* coating. The morphologies and phase compositions of five coatings with different Cr contents were comparatively observed. In addition, the hardness and high-temperature oxidation resistance of the coatings were also analyzed. As a result, with the increase in Cr, the coating grains were more refined. All the coating is mainly composed of the BCC solid-solution phase, which promotes the precipitation of the Laves phase with the increase in Cr. The addition of Cr greatly improves the hardness, high-temperature oxidation resistance and corrosion resistance of the coating. The WVTaTiCr (Cr_1_) exhibited superior mechanical properties, especially in terms of its exceptional hardness, high-temperature oxidation resistance and outstanding corrosion resistance. The average hardness of the WVTaTiCr alloy coating reaches 627.36 HV. After 50 h of high-temperature oxidation, the oxide weight of WVTaTiCr increases by 5.12 mg/cm^2^, and the oxidation rate is 0.1 mg/(cm^2^·h). In 3.5 wt% NaCl solution, the corrosion potential of WVTaTiCr is −0.3198 V, and the corrosion rate is 0.161 mm/a.

## 1. Introduction

Refractory high-entropy alloys (RHEAs) first appeared in public view in 2010 [1]. Thereafter, they aroused great concern from researchers at home and abroad. Research has proved that RHEAs have excellent mechanical properties at high temperatures, even surpassing the widely used nickel-based alloy [2,3,4,5,6]. RHEAs, with excellent mechanical properties at elevated temperatures, are considered promising high-temperature application materials, which are suitable for complex working conditions such as atomic energy, aerospace, the military industry, advanced nuclear reactors, etc. Previous studies focused on mechanical properties at high- and room-temperatures, but with little research and analysis on high-temperature oxidation resistance [7,8,9]. The research shows that adding the proper amount of Al, Cr, and Si elements into the refractory high-entropy alloy can form a dense oxidation film on the surface of the alloy in a high-temperature environment, thus improving the high-temperature oxidation resistance [10,11,12,13]. Unfortunately, elements Al, Cr, and Si very easily form brittle intermetallic compounds with other refractory elements, resulting in reduced mechanical properties of RHEAs [14,15,16]. As a “candidate” for new high-temperature alloy materials, refractory high-entropy alloys still have top priority in improving high-temperature oxidation resistance.

In recent years, W-based alloys have been widely used in the nuclear energy industry, and W-Ta [17], W-V [18], W-Ti [19], W-Cr [20] have been studied. Research results showed that some W-based binary alloys showed brittle behavior [21]. The reduction in ductility makes it difficult for W-based binary alloys to be widely used in harsh working environments, which also urges us to shift our research focus to W- based high-entropy alloys, providing a new idea for the development of heat insulation and radiation protection materials for nuclear reactors. W and Ta elements have adequate mechanical properties and resistance to irradiation-induced embrittlement and swelling [22]. Ti plays a significant role in improving the sintered density [19], whereas V aids in improving the strength and hardness of refractory HEAs [23]. Another refractory metal, Cr was also chosen for use, considering its ability for high-temperature oxidation [24,25,26,27]. The high-entropy alloys undergo segregation when prepared by melting, and embrittlement and porosity are attributes of conventional sintering, whereas the low thickness of the final product is a limit of the physical vapor deposition (PVD) approach [28]. In addition, electrodeposition techniques lead to pores and microcracks in the coating [29]. With the progress of science and technology and the continuous optimization of process technology, laser cladding technology has gradually appeared in public view and has become a hot research topic [30]. Laser cladding, an advanced surface modification technology, cures metal powder into the substrate or chosen material surface, utilizing a high-power laser [31,32,33]. Compared with magnetron sputtering, PTA welding, and additive manufacturing, the research shows that the cladding layer forms firm metallurgical bonds with the substrate, the heat-affected zone of the coating is small, the deformation of the substrate is small, and the coating thickness can reach the millimeter level [30]. Based on comprehensive consideration, W, V, Ta, Ti, and Cr elements are used to develop a new refractory high-entropy alloy with both hardness and high-temperature oxidation resistance. In this study, WVTaTiCr*_x_* RHEAs coating was prepared on the 42-CrMo steel surface using laser cladding, and the trends in the coating microstructure, hardness, high-temperature oxidation resistance, and corrosion resistance were analyzed by varying the Cr content.

## 2. Materials and Methods

The 42-CrMo steel was selected as the substrate. The laser cladding powder was W, V, Ta, Ti, and Cr, with the particle size of 45–105μm and powder purity of 99.9%. Table 1 shows the basic properties of the five elements. Five kinds of powder were mixed using ball milling, in a specific proportion. The mass fraction of each element is shown in Table 2, abbreviated as Cr_0_, Cr_0.25_, Cr_0.5_, Cr_0.75_, and Cr_1_ for the five alloy coatings. The powder was mixed fully in a planetary ball mill. The rotating speed of the ball mill was 300 r/min, and the ball-to-material ratio was 10:1. After 24 h of uninterrupted ball milling, the powder was dried and stored under a vacuum.

This cladding experiment was completed on the LYHS series ultra-high-speed laser-cladding machine tool, whose workbench can move the X-Y-Z three axes with multiple degrees of freedom. In this experiment, the presetting method of coating was used, and argon gas was used as a protective gas. After several parameter adjustments, the optimum process parameters were as follows: the laser power was 1.4 kw, the scanning speed was 8 mm/s, the spot diameter was 6 mm, and the overlap rate was 30–40%.

After cladding, the coating surfaces were polished with sandpaper, then corroded with aqua regia (the ratio of concentrated nitric acid to the concentrated hydrochloric acid solution was 1:3), and cleaned with absolute ethanol. A scanning electron microscope (SEM, Phenom ProX, Phenom-World, Eindhoven, The Netherlands) and its accompanying energy dispersive analyzer (EDS) were used to observe and measure the surface morphology, metallographic structure, and chemical composition content of the coatings. The Smart Lab X-ray diffractometer (Rigaku, Tokyo, Japan) (anode Cu target, X-ray Kα, X-ray wavelength 0.1542 nm, and scanning rate ranging from 1°/min to 20°/min) was used for phase analysis of the coating.

The hardness changes in the samples were analyzed with HXD-1000TMC microhardness tester under a load of 1.96 N and a loading time of 15 s. Hardness tests were carried out every 0.1 mm along the vertical direction of the interface between the substrate and the cladding layer, and the hardness changes in the coatings were recorded. The original quality of the sample was weighed with an electronic balance (accuracy 0.0001 g) and recorded. The sample was put into the corundum ceramic boat, the ceramic boat was placed in the center of the muffle furnace, and the heating rate set at 5 °C/min and the target temperature at 800 °C. After taking out the sample every 10 h, it was air-cooled quickly, weighed, and the quality change recorded. The whole high-temperature oxidation experiment lasted 50 h. A dynamic polarization electrochemical test in the 3.5 wt% NaCl aqueous solution was conducted with a potentiostat (Gamary Interface 1000, Warminster, PA, USA) to evaluate the corrosion behaviors of the RHEA coatings. The cladding layer was the working electrode, and the auxiliary and reference electrodes were platinum and saturated calomel, respectively. The corrosion current density (I_corr_), corrosion potential (E_corr_), and corrosion rate (V) were determined using the Tafel analysis method.

## 3. Results and Discussion

### 3.1. Microstructure Characterization

**Figure 1:**
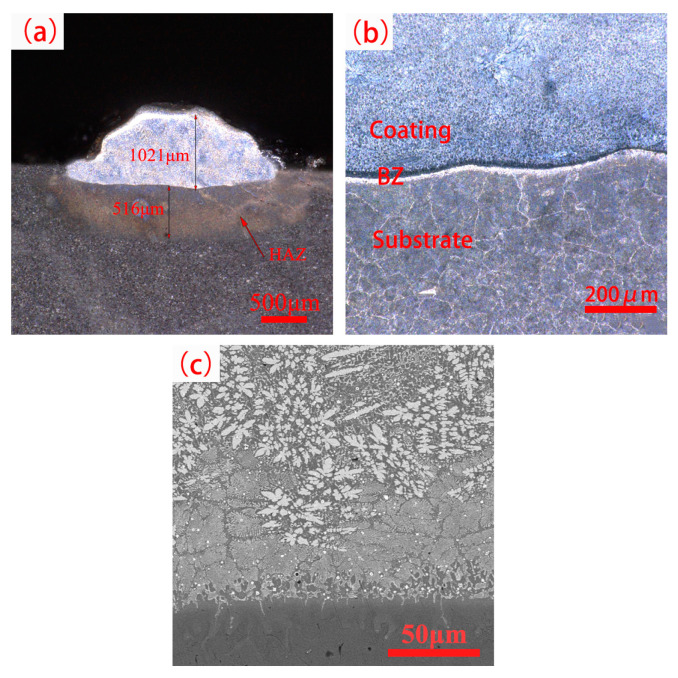
The cladding condition of the coating cross-section. (**a**) coating cross-section; (**b**) the binding zone at low multiples; (**c**) the binding zone at high multiples.

WVTaTiCr*_x_* (*x* = 0, 0.25, 0.5, 0.75, 1) RHEA coatings were prepared under the optimum process parameters, and the cladding condition of the coating cross section was observed for WVTaTi(Cr_0_) as an example, and the results are shown in Figure 1. From Figure 1a, it is observed that the thickness of the coatings is about 1 mm and the thickness of the heat-affected zone (HAZ) is 0.3~0.5 mm. From Figure 1b,c, it is seen that the metallurgical bonding between the 42CrMo steel and the coating is good, and there are no obvious cracks and pores near the bonding zone (BZ). In addition, the typical dendrite structures are formed near the interface between the coating and the substrate, and grow perpendicular to the boundary.

**Figure 2:**
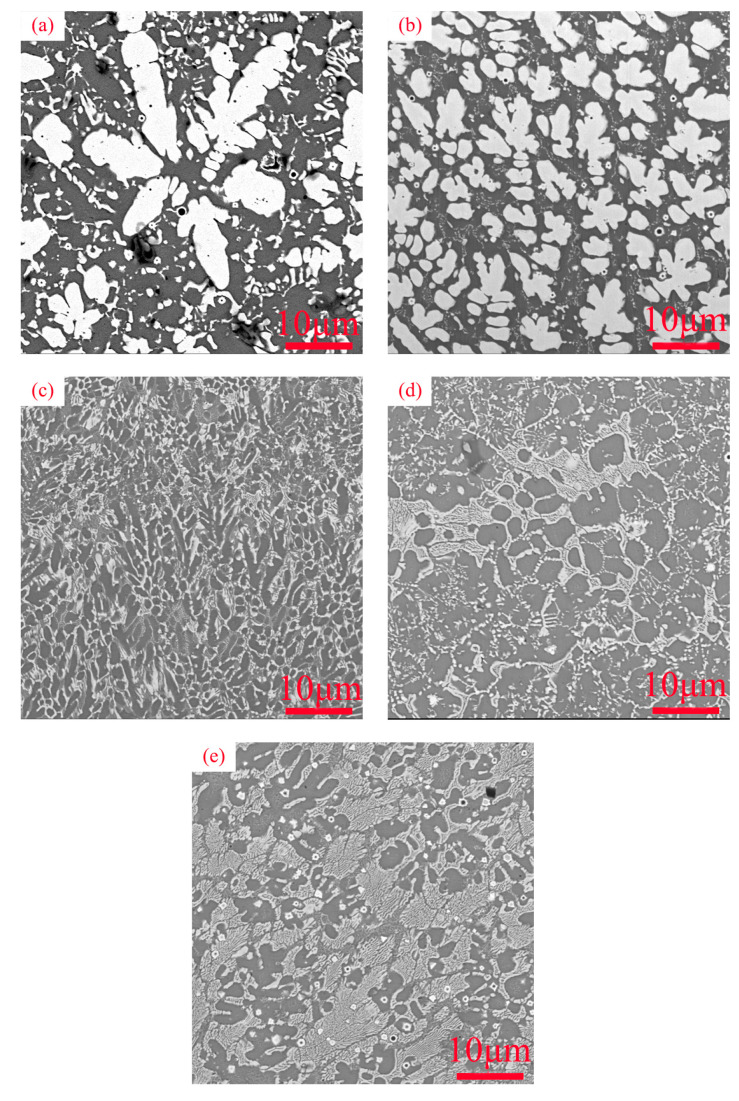
SEM of the cross-section of WVTaTiCr*_x_* refractory high-entropy alloy coating: (**a**) Cr_0_; (**b**) Cr_0.25_; (**c**) Cr_0.5_; (**d**) Cr_0.75_; (**e**) Cr_1_.

Figure 2 shows the microscopic morphology of different coatings. It can be seen from the diagram that the structural morphology of the cladding layer changes obviously with the change in element content. As shown in Figure 2a,b, when there is no, or only a small amount of, Cr in the coating, the coating mainly consists of white petal-like dendrites and a gray base. As the elemental Cr was added to the coating, the dendritic structure became uniform. From Figure 2d, it can be seen that feather-like structures were generated in the coating. In Figure 2e, it is observed that the structure of Cr_1_ refines and the area of feather-like tissue increases, showing a network distribution within the dendritic region.

**Figure 3:**
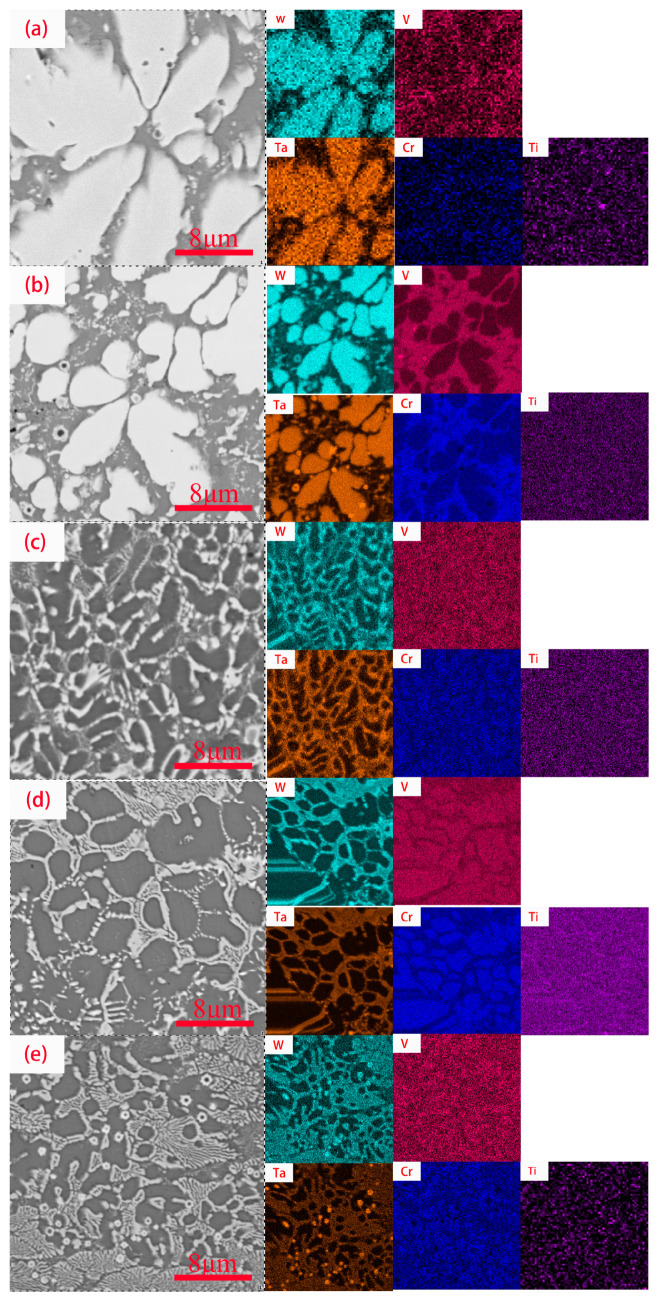
EDS of cross section of WVTaTiCr*_x_* RHEA coating: (**a**) Cr_0_; (**b**) Cr_0.25_; (**c**) Cr_0.5_; (**d**) Cr_0.75_; (**e**) Cr_1_.

Figure 3 shows the EDS element distribution of the WVTaTiCr*_x_* RHEA coating. The figure shows that during the solidification process of the alloy powder, the distribution of elements in the cladding layer is not uniform. Ta and W with high melting points are first solidified and formed, and mainly distributed in the white dendrite area; there is a high content of V and Cr elements in the gray area, and Ti is uniformly distributed in the alloy. In addition, the minimum enthalpy of mixing (−7 KJ/mol) of Cr and Ta means that during the solidification process, Cr and Ta are more easily combined to form intermetallic compounds, which leads to the formation of Cr_2_Ta in the Laves phase. It is evident from Figure 3e that the feathered Laves phase distribution forms a network with white dendritic areas. In addition, the presence of Cr was also detected in Figure 3a. As the laser cladding makes the 42-CrMo steel dilute, the Cr element in the substrate penetrates the coating.

### 3.2. Phase Analysis

**Figure 4:**
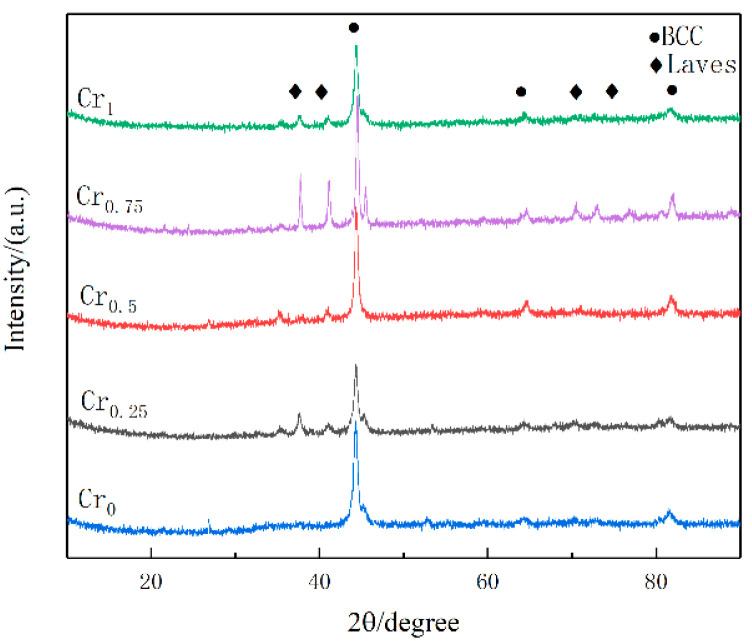
XRD of WVTaTiCr*_x_* high entropy alloy coating.

Phase analysis of five high-entropic alloy-coated samples with different contents of the Cr element was carried out, and the phase in the alloy was relatively simple. The main refractory metal elements are the BCC phase, so refractory high-entropy alloys are mostly simple BCC solid-solution phase, and sometimes the second phase is precipitated [35]. As shown in Figure 4, the positions of the diffraction peaks of the four components are approximately the same, consisting of the main peak of one (110) crystal plane and the low peak of (200) (211) crystal planes, mainly concentrated at the diffraction angles of 44.484°, 64.777° and 81.983°. Compared with the PDF card (PDF#34-0396), WVTaTiCr*_x_* RHEA coatings are based on the BCC solid-solution phase, and the addition of Cr promotes the precipitation of the Laves phase (Cr_2_Ta), because the addition of Cr causes more severe lattice distortion in the alloy, thus opening a path for atomic diffusion [36]. At the same time, the enthalpy of mixing formed between Cr and other refractory elements is small, which reduces the enthalpy value in the alloy and the stability of the BCC phase, which leads to the precipitation of the Laves phase. From the thermodynamic point of view, if the enthalpy of mixing between the elements is positive, then the atoms of the two elements tend to repel each other, whereas the atoms of the two elements tend to attract each other to form a stable phase. The atomic radius of the Cr atom is the smallest and the easiest to diffuse, and the enthalpy of mixing between Cr and Ta is −7 KJ/mol. The binding ability between Cr and Ta is strong, and it is easier to form Cr_2_Ta, which can be precipitated as the Laves phase. There are no other complex intermetallic compounds in the figure, so the high-entropy effect effectively inhibits the precipitation of intermetallic compounds.

### 3.3. Hardness

**Figure 5:**
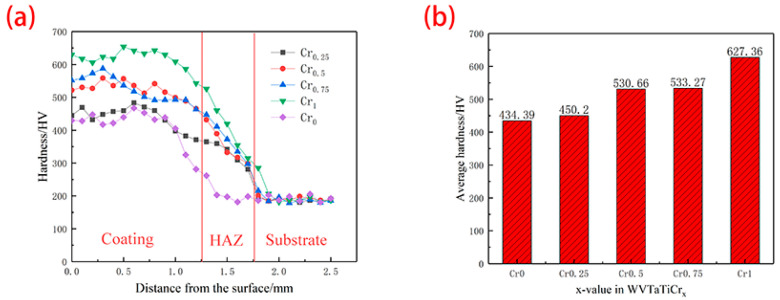
Hardness of WVTaTiCr*_x_* RHEA coating: (**a**) cross-section hardness; (**b**) average hardness of coating area.

Figure 5 summarizes the trend in hardness change of the cladding samples with different Cr contents. Figure 5a depicts the hardness distribution of the cross-section of high-entropy alloy coatings, and Figure 5b depicts the average hardness of high-entropic alloy coatings. As can be seen from Figure 5a, the entire cross-section is divided into three areas, namely, coating, heat-affected zone (HAZ), and substrate. The hardness of the whole coating area is significantly higher than that of the substrate. This is mainly because the laser cladding technology outputs a lot of heat momentarily through a high-energy laser beam, and the pre-set coating melts and solidifies rapidly, which produces a large supercooling degree during solidification, increases the nucleation rate, promotes a shorter growth time for each dendrite, collides with other grains, and grows again. The effect of grain refinement is significantly enhanced; thus, the hardness of the coating is generally higher than that of the substrate. As can be seen from Figure 5b, with the increase in Cr content, the average hardness of the coating also increases. According to previous studies, the hardness value of alloys has a strong relationship with the elements and phase composition. Combining XRD with SEM analysis, with the increase in Cr content, the Laves phase precipitation in the alloy also increases, and the precipitation strengthening effect is enhanced. In addition, with the increase in Cr content, the solid-solution strengthening effect in the alloy is enhanced, which further increases the hardness.

### 3.4. Oxidation Resistance

**Figure 6:**
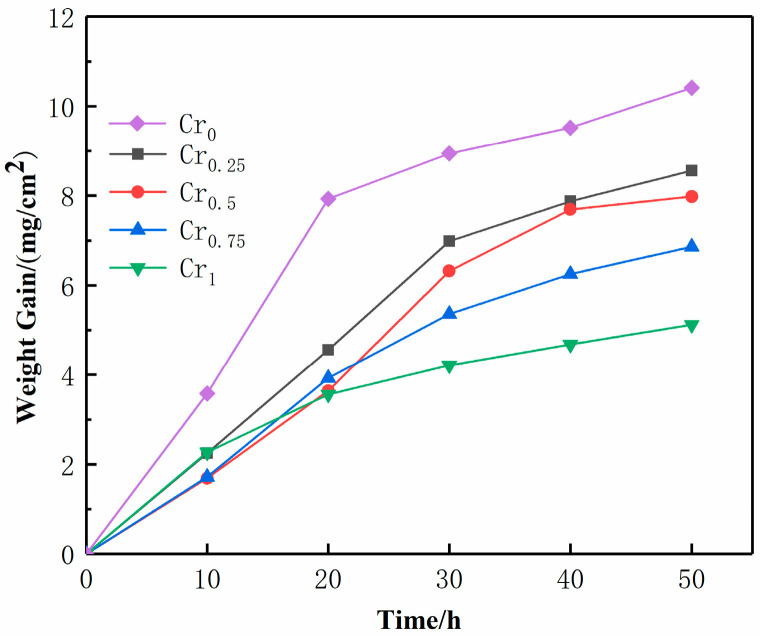
Weight increase of oxide on the surface of WVTaTiCr*_x_* RHEA coating.

Figure 6 summarizes the trend of oxide weight gain on different component coating surfaces after 50 h continuous oxidation of laser cladding samples at 800 °C. It can be seen from Figure 6 that, after adding the Cr element based on WVTaTi (Cr_0_), the oxide weight gain decreases significantly. With the increase in oxidation time, the oxide weight-gain curve has continuity, and the content of Cr in the coating will affect the overall high-temperature oxidation resistance. In the first half of the experiment, the rate of oxidation weight increase is very fast, while, on the contrary, the rate of oxidation weight increase is slow after 20 h. Previous studies have shown that the high content of Cr in high-entropy alloys and the slow diffusion effect of high-entropy alloys can improve the high-temperature oxidation resistance of high-entropy alloys [12,37,38].

Weight gain (mg/cm^2^) and weight-gain rate (mg/(cm^2^·h)) of WVTaTiCr*_x_* RHEA coating after oxidation at 800 °C for 50 h are shown in Table 3. When Cr is not added, the weight gain and oxidation rate of the WVTaTi high-entropy alloy coating oxide are 10.41 mg/cm^2^ and 0.21 mg/(cm^2^·h), respectively. With the increase in Cr content, the weight increases of oxide on the coating surface decreases. The addition of Cr resulted in the smallest increase in oxidation weight of the Cr_1_ RHEA coating, with a mass increase of 5.12 mg/cm^2^ and an oxidation rate of 0.10 mg/(cm^2^·h). According to the oxidation-resistance rating standard of steel, superalloy, and high-temperature protective coatings (HB5258-2000) [39], it can be seen that Cr_1_ RHEA coating belongs to the antioxidant alloys, and Cr_0.25_, Cr_0.5_, and Cr_0.75_ belong to the sub-antioxidant alloys. In general, the proper addition of the Cr element can effectively improve the oxidation resistance of the coating.

**Figure 7:**
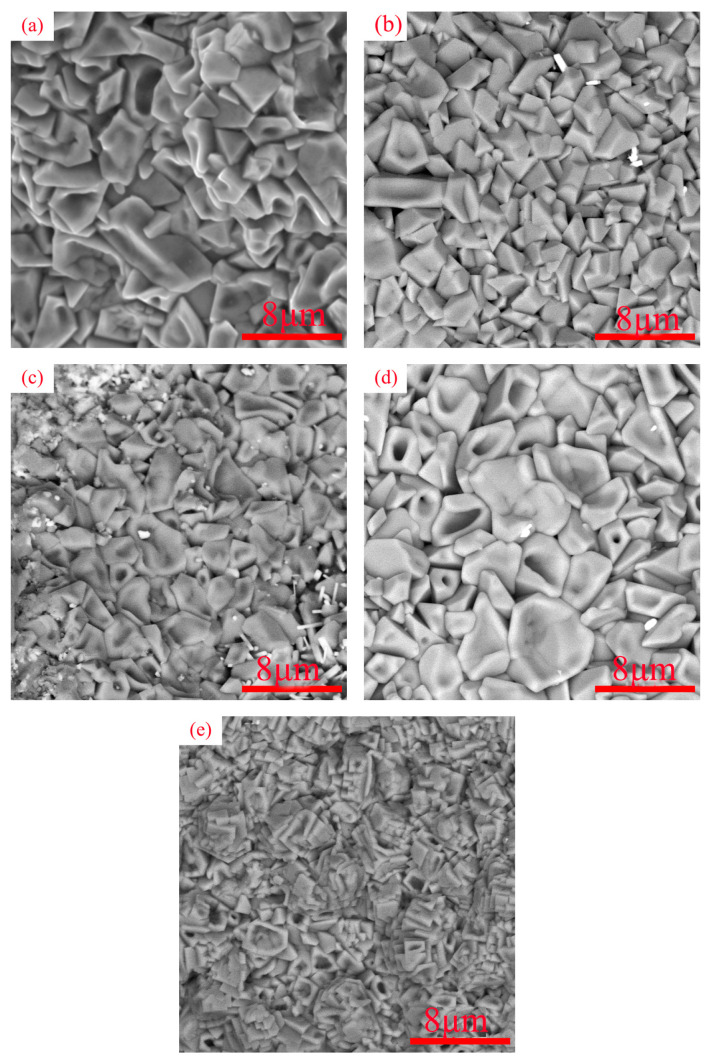
SEM of surface oxidation of WVTaTiCr*_x_* RHEA coating: (**a**) Cr_0_; (**b**) Cr_0.25_; (**c**) Cr_0.5_; (**d**) Cr_0.75_; (**e**) Cr_1_.

**Figure 8:**
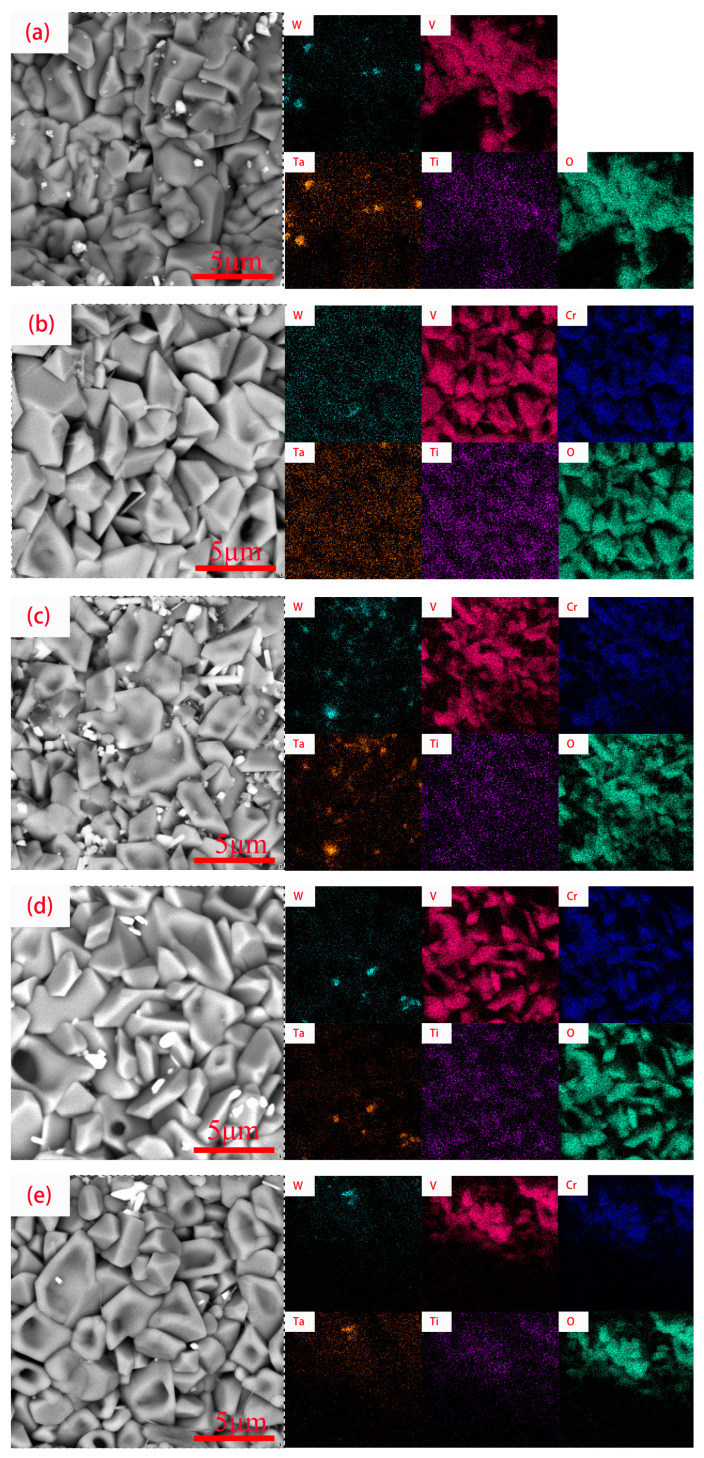
EDS element analysis of oxidation surface of WVTaTiCr*_x_* RHEA coating: (**a**) Cr_0_; (**b**) Cr_0.25_; (**c**) Cr_0.5_; (**d**) Cr_0.75_; (**e**) Cr_1_.

Figure 7 shows the surface micromorphology of the coating of WVTaTiCr*_x_* RHEA after oxidation at 800 °C for 50 h. As can be seen from the figure, the coating surface generates large areas of irregular polygonal blocky oxides, which are overlapped with different sizes. To intuitively observe the distribution of elements, surface scanning analysis was performed on the selected areas of the oxidized surface, and the results are shown in Figure 8. From the figure, it can be seen that with the high-temperature oxidation in the same environment, the elemental distribution on the surface of the five coatings is approximately the same, and it can also be judged that the oxides on the surface are also basically similar. EDS showed that the oxygen content in the dendrite was greater than the interdendritic region, indicating that the oxidation resistance of the dendrites was weaker than the interdendritic region. V, Cr, Ti, and O are enriched elements, mainly concentrated in interdendritic regions; the proportion of W and Ta content on the entire surface is small. Moreover, as shown in Figure 3a, the dilution of laser cladding technology allows Cr from 42CrMo steel to diffuse into the WVTaTi coating. However, due to the small amount of Cr content, it cannot be detected after high-temperature oxidation.

**Figure 9:**
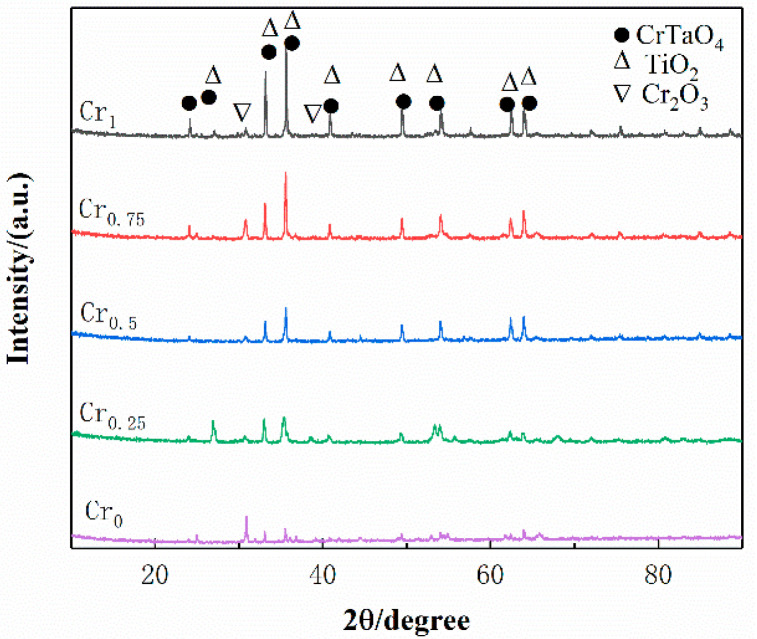
Surface oxide XRD of WVTaTiCr*_x_* RHEA coating.

To explore the types of oxides, XRD analysis was performed on the surface of the coatings after oxidation. As shown in Figure 9, the oxide mainly consists of CrTaO_4_, TiO_2,_ and Cr_2_O_3_; the Cr_1_ alloy has the highest oxide diffraction peak, indicating that more oxides are generated, making the oxide film on the coating surface uniform and dense. The dense oxide film inhibits the diffusion of oxygen atoms, reduces the oxidation rate, and improves oxidation resistance. This conclusion is also consistent with the results presented by the oxide weight-gain curve. The results show that adding Cr to the WVTaTi alloy can effectively improve high-temperature oxidation resistance.

Table 4 shows the standard Gibbs free energy of molar formation of the oxides of Cr, Ta, and Ti at 800 °C. Due to the smallest Gibbs free energy of TiO_2_, it is first generated and exists stably. The Gibbs free energy of Ta_2_O_5_ is −598 KJ/mol, which is the second smallest, after the TiO_2_. Due to the atomic radius of Ta being slightly larger than Cr and Ti, the diffusion rate is relatively slow. In addition, there is a lattice distortion effect inside high-entropy alloys, and atomic diffusion needs to overcome varying resistance. This slow diffusion effect makes it more difficult for Ta to generate oxides, so Ta_2_O_5_ does not appear on the surface. Although the standard Gibbs free energy of molar formation of Cr_2_O_3_ is greater than Ta_2_O_5_, the diffusion rate of Cr is faster than Ta, which also contributes to the formation of Cr_2_O_3_. Research shows that the Cr and Ta elements have a strong binding capacity with oxygen, so a small amount of CrTaO_4_ can also be generated. With the addition of Cr, the internal lattice distortion effect of the WVTaTiCr alloy is the strongest, and the diffusion rate of each atom is the slowest, resulting in a decrease in the binding speed with external oxygen elements. This conclusion confirms the oxide XRD patterns and oxidation kinetic curves. The addition of Cr makes atomic diffusion slow, and the oxidation rate and weight-gain decrease, thereby improving the high-temperature oxidation resistance of the alloy.

### 3.5. Corrosion Resistance

**Figure 10:**
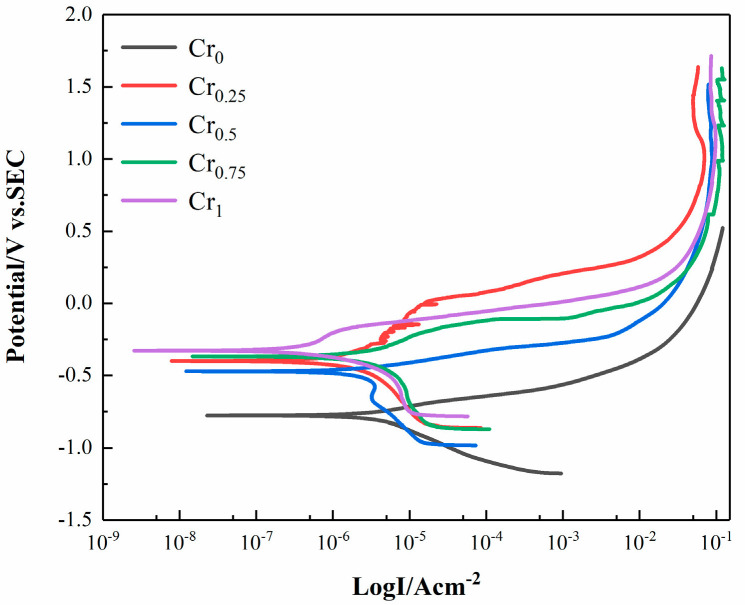
Tafel curve of WVTaTiCr*_x_* RHEA coating in 3.5%wt NaCl solution.

The Tafel curves of each coating surface in 3.5 wt% NaCl solution are shown in Figure 10. Table 5 summarizes the corrosion potential (E_corr_), corrosion current density (I_corr_), and corrosion rate (V) in the Tafel curves of each coating. According to Figure 10, the self-corrosion potential of the WVTaTi (Cr_0_) alloy is the lowest, and the self-corrosion potential of the alloy gradually increases with the addition of Cr. Among these, the WVTaCrTi (Cr_1_) alloy coating has the highest corrosion potential. The data can be derived from Table 5; the corrosion potential and corrosion rate of WVTaTi (Cr_0_) are −0.7783 V and 2.959 mm/a, respectively, and the corrosion potential and corrosion rate of the WVTaCrTi (Cr_1_) alloy coating are −0.3198 V and 0.161 mm/a, respectively. The addition of Cr can promote the generation of the passive film on the surface of the alloy coating, while the oxide deposition of Cr can fill the vacancy of the passive film, making the surface passive film become denser and uniform, thus reducing the diffusion and penetration rate of ions in the NaCl solution, decreasing the corrosion rate and improving the corrosion resistance of the alloy surface coating.

## 4. Conclusions

The WVTaTiCr*_x_* (*x* = 0, 0.25, 0.5, 0.75, 1) RHEA coating was prepared using laser cladding on the 42-CrMo steel surface. The effect of the Cr content on the microstructure and mechanical properties of the WVTaTiCr*_x_* (*x* = 0, 0.25, 0.5, 0.75, 1) alloy was studied. The analysis results are as follows:

(1) Laser-cladding-forming figures showed good metallurgical bonding between the coatings and the substrate, a uniform and dense coating structure, and a coating thickness of about 1 mm. The results indicate that laser cladding is feasible for the preparation of RHEA coatings.
(2) The WVTaTi (Cr_0_) alloy coating consists of the BCC phase, and the addition of Cr promotes the precipitation of the Laves, which is uniformly distributed in the structure. The WVTaTiCr (Cr_1_) exhibits superior mechanical properties, particularly in terms of its exceptional hardness, high-temperature oxidation resistance, and outstanding corrosion resistance.
(3) The successful preparation of WVTaTiCr alloy coating compensates for the performance shortcomings of some W-based binary alloys, providing a new idea for the development of heat insulation and radiation protection materials for nuclear reactors. The improved mechanical and oxidation properties of WVTaTiCr indicate the potential for future use in fusion applications.

## Figures and Tables

**Table 1:** Basic properties of elements in WVTaTiCr*_x_* RHEA coating [34].

Element	W	V	Ta	Ti	Cr
Atomic radius (Å)	1.37	1.32	1.43	1.46	1.25
Melting point (°C)	3407	1890	2996	1660	1857

**Table 2:** The mass fraction of each element of alloy coatings (wt%).

Alloys	Identification	W	V	Ta	Ti	Cr
WVTaTi	Cr_0_	39.65	10.99	39.03	10.33	
WVTaTiCr_0.25_	Cr_0.25_	38.57	10.69	37.97	10.12	2.74
WVTaTiCr_0.5_	Cr_0.5_	37.55	10.40	36.96	9.78	5.31
WVTaTiCr_0.75_	Cr_0.75_	36.58	10.14	36	9.52	7.76
WVTaTiCr_1_	Cr_1_	35.66	9.88	35.09	9.28	10.08

**Table 3:** Weight gain (mg/cm^2^) and weight-gain rate (mg/cm^2^·h) of WVTaTiCr*_x_* RHEA coating after oxidation at 800 °C for 50 h.

Alloys	Cr_0_	Cr_0.25_	Cr_0.5_	Cr_0.75_	Cr_1_
Δm/s	10.41	8.56	7.98	6.86	5.12
Δm/(s·t)	0.21	0.17	0.16	0.14	0.10

**Table 4:** Standard Gibbs free energy of metal oxidation products of Cr, Ta, and Ti at 800 °C.

Oxide	Cr_2_O_3_	TiO_2_	CrTaO_4_	Ta_2_O_5_
ΔG (KJ/mol O_2_)	−538	−713	−568	−598

**Table 5:** Tafel Curve Electrochemical Parameters of WVTaTiCr*_x_* RHEA coating.

Alloys	E_corr_ (V)	I_corr_ (uA/cm^2^)	V (mm/a)
Cr_0_	−0.7783	6.466 × 10^−6^	2.959
Cr_0.25_	−0.4005	6.848 × 10^−6^	2.545
Cr_0.5_	−0.4663	1.059 × 10^−6^	0.394
Cr_0.75_	−0.3692	1.972 × 10^−5^	7.329
Cr_1_	−0.3198	4.337 × 10^−7^	0.161

## Data Availability

The data presented in this study are available from the corresponding author upon reasonable request.

## References

[1] Senkov O.N., Wilks G.B., Miracle D.B., Chuang C.P., Liaw P.K. (2010). Refractory high-entropy alloys. Intermetallics.

[2] Kumar R., Torres H., Aydinyan S., Antonov M., Varga M., Hussainova I., Rodriguez Ripoll M. (2023). Tribological behavior of Ni-based self-lubricating claddings containing sulfide of nickel, copper, or bismuth at temperatures up to 600 °C. Surf. Coat. Technol..

[3] Hector T., Tugce C., Jens H., Janne N., Braham P., Manel R.R. (2022). Tribological performance of iron- and nickel-base self-lubricating claddings containing metal sulfides at high temperature. Friction.

[4] Couzinié J., Dirras G., Perrière L., Chauveau T., Leroy E., Champion Y., Guillot I. (2014). Microstructure of a near-equimolar refractory high-entropy alloy. Mater. Lett..

[5] Juan C.-C., Tsai M.-H., Tsai C.-W., Lin C.-M., Wang W.-R., Yang C.-C., Chen S.-K., Lin S.-J., Yeh J.-W. (2015). Enhanced mechanical properties of HfMoTaTiZr and HfMoNbTaTiZr refractory high-entropy alloys. Intermetallics.

[6] Wu Y.D., Cai Y.H., Wang T., Si J.J., Zhu J., Wang Y.D., Hui X.D. (2014). A Refractory Hf25Nb25Ti25Zr25 High-Entropy Alloy with Excellent Structural Stability and Tensile Properties. Mater. Lett..

[7] Yao H.W., Qiao J.W., Gao M.C., Hawk J.A., Ma S.G., Zhou H.F., Zhang Y. (2016). NbTaV-(Ti, W) Refractory High-entropy Alloys: Experiments and Modeling. Mater. Sci. Eng. A.

[8] Zhang H., Zhao Y., Cai J., Ji S., Geng J., Sun X., Li D. (2021). High-strength NbMoTaX refractory high-entropy alloy with low stacking fault energy eutectic phase via laser additive manufacturing. Mater. Des..

[9] Zhang Y., Yi-Wen Z., Feng-Ge Z., Tao Y., Chen X.C. (2003). Effect of Powder Particle Size on Microstructure and Mechanical Property of Ni-Based P/M Superalloy Product. J. Iron Steel Res..

[10] Ding X., He J., Zhong J., Wang X., Li Z., Tian J., Dai P. (2022). Effect of Al Addition on Microstructure and Properties of CoCrNi Medium-Entropy Alloy Prepared by Powder Metallurgy. Materials.

[11] Thürlová H., Průša F. (2022). Influence of the Al Content on the Properties of Mechanically Alloyed CoCrFeNiMn_X_Al_20−X_ High-Entropy Alloys. Materials.

[12] Kumar T.S., Sourav A., Murty B., Chelvane A., Thangaraju S. (2022). Role of Al and Cr on cyclic oxidation behavior of AlCoCrFeNi_2_ high entropy alloy. J. Alloys Compd..

[13] Chen L., Zhou Z., Tan Z., He D., Bobzin K., Zhao L., Öte M., Königstein T. (2018). High temperature oxidation behavior of Al_0.6_CrFeCoNi and Al_0.6_CrFeCoNiSi_0.3_ high entropy alloys. J. Alloys Compd..

[14] Jiang Y., Zhao N., Peng L.L., Zhao L.N., Liu M. (2016). Microstructure and mechanical properties at elevated temperatures of a new Al-containing refractory high-entropy alloy Nb-Mo-Cr-Ti-Al. J. Alloys Compd..

[15] Qin Y., Liu J.X., Li F., Wei X., Wu H., Zhang G.J. (2019). A high entropy silicide by reactive spark plasma sintering. J. Adv. Ceram..

[16] Yurchenko N., Stepanov N., Gridneva A., Mishunin M., Salishchev G., Zherebtsov S. (2018). Effect of Cr and Zr on phase stability of refractory Al-Cr-Nb-Ti-V-Zr high-entropy alloys. J. Alloys Compd..

[17] Mooney H.A., Cropper A., Reid W. (2016). Effects of grain size on the microstructure and texture of cold-rolled Ta-2.5W alloy. Int. J. Refract. Met. Hard Mater..

[18] Arshad K., Guo W., Wang J., Zhao M.-Y., Yuan Y., Zhang Y., Wang B., Zhou Z.-J., Lu G.-H. (2015). Influence of vanadium precursor powder size on microstructures and properties of W–V alloy. Int. J. Refract. Met. Hard Mater..

[19] Dai W., Liang S., Luo Y., Yang Q. (2015). Effect of W powders characteristics on the Ti-rich phase and properties of W–10 wt.% Ti alloy. Int. J. Refract. Met. Hard Mater..

[20] Ma Y., Han Q.-F., Zhou Z.-Y., Liu Y.-L. (2016). First-principles investigation on mechanical behaviors of W–Cr/Ti binary alloys. J. Nucl. Mater..

[21] Rieth M., Dudarev S.L., De Vicente S.G., Aktaa J., Ahlgren T., Antusch S., Armstrong D.E.J., Balden M., Baluc N., Barthe M.F. (2013). Recent progress in research on tungsten materials for nuclear fusion applications in Europe. J. Nucl. Mater..

[22] Xiao X., Liu G., Hu B., Wang J., Ma W. (2015). Microstructure Stability of V and Ta Microalloyed 12%Cr Reduced Activation Ferrite/Martensite Steel during Long-term Aging at 650 °C. J. Mater. Sci. Technol..

[23] Senkov O.N., Wilks G.B., Scott J.M., Miracle D.B. (2011). Mechanical properties of Nb_25_Mo_25_Ta_25_W_25_ and V_20_Nb_20_Mo_20_Ta_20_W_20_ refractory high entropy alloys. Intermetallics.

[24] Jin X., Liang Y., Bi J., Li B. (2019). Non-monotonic variation of structural and tensile properties with Cr content in AlCoCr_x_FeNi_2_ high entropy alloys. J. Alloys Compd..

[25] Yan X., Guo H., Yang W., Pang S., Wang Q., Liu Y., Liaw P.K., Zhang T. (2021). Al_0_._3_Cr_x_FeCoNi high-entropy alloys with high corrosion resistance and good mechanical properties—ScienceDirect. J. Alloys Compd..

[26] Ben Q., Zhang Y., Sun L., Wang L., Wang Y., Zhan X. (2022). Wear and Corrosion Resistance of FeCoCr_x_NiAl High-Entropy Alloy Coatings Fabricated by Laser Cladding on Q345 Welded Joint. Metals.

[27] Senkov O.N., Senkova S.V., Woodward C., Miracle D.B. (2013). Low-density, refractory multi-principal element alloys of the Cr–Nb–Ti–V–Zr system: Microstructure and phase analysis. Acta Mater..

[28] Waseem O.A., Ryu H.J. (2017). Powder Metallurgy Processing of a W_x_TaTiVCr High-Entropy Alloy and Its Derivative Alloys for Fusion Material Applications. Sci. Rep..

[29] Moshtaghi M., Safyari M., Mori G. (2022). Hydrogen absorption rate and hydrogen diffusion in a ferritic steel coated with a micro- or nanostructured ZnNi coating. Electrochem. Commun..

[30] Erario M.d.l.Á., Croce E., Moviglia Brandolino M.T., Moviglia G., Grangeat A.M. (2021). A review on laser cladding of high-entropy alloys, their recent trends and potential applications. J. Manuf. Process..

[31] Liu H., Gao Q., Dai J., Chen P., Gao W., Hao J., Yang H. (2022). Microstructure and high-temperature wear behavior of CoCrFeNiWx high-entropy alloy coatings fabricated by laser cladding. Tribol. Int..

[32] Zhou J., Kong D. (2021). Friction–Wear performances and oxidation behaviors of Ti3AlC2 reinforced Co–based alloy coatings by laser cladding. Surf. Coat. Technol..

[33] Qiu X.-W., Liu C.-G. (2013). Microstructure and properties of Al2CrFeCoCuTiNix high-entropy alloys prepared by laser cladding. J. Alloys Compd..

[34] Zhou J.-L., Cheng Y.-H., Chen Y.-X., Liang X.-B. (2022). Composition design and preparation process of refractory high-entropy alloys: A review. Int. J. Refract. Met. Hard Mater..

[35] Senkov O.N., Scott J.M., Senkova S.V., Miracle D.B., Woodward C.F. (2011). Microstructure and room temperature properties of a high-entropy TaNbHfZrTi alloy. J. Alloys Compd..

[36] Gao X., Chen R., Liu T., Fang H., Wang L., Su Y. (2022). High deformation ability induced by phase transformation through adjusting Cr content in Co-Fe-Ni-Cr high entropy alloys. J. Alloys Compd..

[37] Holcomb G.R., Tylczak J.H., Carney C.S. (2015). Oxidation of CoCrFeMnNi High Entropy Alloys. Jom.

[38] Zhang Y., Wu H., Yu X., Tang D. (2022). Role of Cr in the High-Temperature Oxidation Behavior of Cr_x_MnFeNi High-Entropy Alloys at 800 °C in Air. Soc. Sci. Electron. Publ..

[39] Wang H., Tang Q., Li X., Dai P. (2017). The effect of N on the oxidation resistance of CoCrFeMnNi high entropy alloy. Met. Heat Treat..

